# Effects of Misalignments in the Retinal Nerve Fiber Layer Thickness Measurements with Spectral Domain Optical Coherence Tomography

**DOI:** 10.1155/2014/412915

**Published:** 2014-12-09

**Authors:** Kleyton A. Barella, Fernanda Cremasco, Camila Zangalli, Vital P. Costa

**Affiliations:** Faculty of Medical Sciences, State University of Campinas (UNICAMP), Rua Vital Brasil 251, 13083-888 Campinas, SP, Brazil

## Abstract

*Purpose*. To investigate misalignments (MAs) on retinal nerve fiber layer thickness (RNFLT) measurements obtained with Cirrus^©^ SD-OCT. *Methods*. This was a retrospective, observational, cross-sectional study. Twenty-seven healthy and 29 glaucomatous eyes of 56 individuals with one normal exam and another showing MA were included. MAs were defined as an improper alignment of vertical vessels in the en face image. MAs were classified in complete MA (CMA) and partial MA (PMA), according to their site: 1 (superior, outside the measurement ring (MR)), 2 (superior, within MR), 3 (inferior, within MR), and 4 (inferior, outside MR). We compared RNFLT measurements of aligned versus misaligned exams in all 4 sectors, in the superior area (sectors 1 + 2), inferior area (sectors 3 + 4), and within the measurement ring (sectors 2 + 3). *Results*. RNFLT measurements at 12 clock-hour of eyes with MAs in the superior area (sectors 1 + 2) were significantly lower than those obtained in the same eyes without MAs (*P* = 0.043). No significant difference was found in other areas (sectors 1 + 2 + 3 + 4, sectors 3 + 4, and sectors 2 + 3). *Conclusion*. SD-OCT scans with superior MAs may present lower superior RNFLT measurements compared to aligned exams.

## 1. Introduction

Glaucoma is a progressive optic neuropathy characterized by death of retinal ganglion cells (RGC) and degeneration of the retinal nerve fiber layer (RNFL), resulting in a distinct appearance of the optic nerve head (ONH) and concomitant visual field (VF) loss [1]. However, in the early stages of the disease, structural changes may precede VF defects. Some studies have shown that as many as half of RGC can be lost before a VF defect is detected by standard automated perimetry (SAP) [2, 3]. Therefore, detecting structural changes in the neuroretinal rim and RNFL is important for early glaucoma detection.

Several techniques have been introduced over the past years aiming at detecting morphologic glaucomatous abnormalities earlier than functional tests [4]. First described by Huang et al., 1991 [5], optical coherence tomography (OCT) has been widely accepted in glaucoma management [6]. The Cirrus (Carl Zeiss Meditec Inc., Dublin, CA) spectral domain OCT (SD-OCT) offers greater image axial resolution (5 *μ*m) and faster acquisition speeds (27,000 A-scans per second) when compared to previous generations time domain OCTs (TD-OCTs) [7].

However, even with a faster image acquisition (approximately 2 seconds), RNFL thickness (RNFLT) measurements may be affected by artifacts and misalignments (MAs), which may occur as a result of eye or head movements [8]. In a recent study [9], Moreno-Montañés et al. evaluated the frequency of MAs in normal individuals and individuals with glaucoma imaged with the Cirrus SD-OCT and observed whether these misalignments resulted in changes in RNFLT measurements. The authors found a high frequency of MAs and no difference in RNFLT measurements when they compared scans showing complete MAs (CMAs) and scans without CMAs. However, the effects of the CMA were analyzed in all patients regardless of the site of MA and exams were obtained in different days. We hypothesize that MAs detected in a given sector will be more prone to affect measurements obtained in that same sector. Hence, the purpose of the present study was to investigate the effects of MAs on RNFLT measurements according to the site of the MA.

## 2. Methods

### 2.1. Subjects

This was a retrospective, observational, cross-sectional study. The study was approved by the University of Campinas Medical Institutional Review Board and followed the tenets of the Declaration of Helsinki. Participants in this study were included in a previously published study by our group [10]. One randomly selected eye from 34 patients with glaucoma and 32 healthy individuals was included in that study. All subjects were recruited from the Glaucoma Service, University of Campinas (UNICAMP), Brazil.

Subjects underwent a complete ophthalmic evaluation that included medical history, best corrected visual acuity (BCVA), slit lamp biomicroscopy, measurement of intraocular pressure (IOP) with Goldmann tonometry, gonioscopy, slit lamp fundus examination with a 78-diopter lens, SAP using the standard 30-2 or 24-2 Swedish interactive threshold algorithm (SITA) or standard full threshold mode (Humphrey Field Analyzer II, Carl Zeiss Meditec Inc., Dublin, CA), and imaging with Cirrus SD-OCT (Carl Zeiss Meditec Inc., Dublin, CA).

In the present study, we included all patients who had at least 1 OCT exam showing only one MA and another exam without MA acquired in the same session. Only one eye per patient was included in the study. In patients with more than one exam with MA, the first image obtained was selected for comparison. Among the 66 patients examined in our previous study, we found 56 patients (29 patients with glaucoma and 27 healthy individuals) with 1 aligned and 1 misaligned exam.

The inclusion criteria for healthy eyes (selected among the staff or patients' spouses) were intraocular pressure (IOP) ≤ 21 mmHg with no history of elevated IOP or glaucoma cases in the family, reliable normal visual field, open angle at gonioscopy and normal optic disc appearance based on clinical stereoscopic examination, no history of ocular or systemic disease or surgery that might interfere with RNFLT measurements, ability to perform the tests, and willingness to participate as a subject in the study.

Subjects in the glaucoma group included those with any form of chronic glaucoma, defined as the presence of optic disc abnormalities consistent with glaucomatous optic neuropathy with or without visual field loss. Two of the following optic disc abnormalities had to be present for the disc to be characterized as glaucomatous: cup/disc ratio > 0.6, localized rim loss, optic disc hemorrhage, or cup/disc asymmetry > 0.3. Optic discs with excessive paleness or accompanied by retinal lesions were excluded.

Exclusion criteria for both groups included age < 18, visual acuity (VA) worse than 20/40, spherical refraction greater than ±5.0 diopters (D), cylinder correction greater than ±3.0 D and unreliable SAPs with false-positive errors >33%, false-negative errors > 33%, and fixation losses > 20%, significant cataract according to the criteria of Lens Opacification Classification System III (LOCSIII), defined as nuclear opacity equal to or greater than NC3 or NO3, cortical equal to or greater than C3, and/or subcapsular equal to or greater than P3 [11], corneal diseases, contact lens use, history of posterior segment intraocular surgery, and systemic or ocular diseases that can cause visual field loss.

### 2.2. Optical Coherence Tomography

RNFLT measurements were obtained with the Cirrus SD-OCT (software version 3.0.0.64) (Carl Zeiss Meditec Inc., Dublin, CA). The ONH mode, which consists of a 3-dimensional dataset of 200 A-scans that are derived from 200 B-scans and analyze a 6 mm^2^ area centered on the optic disc, was utilized. The software generates a RNFL thickness map from the 3-dimensional cube data set centered on the disc. Subsequently, it also extracts RNFLT measurements from a circumpapillary circle of 1.73 mm of radius. RNFLT measurements are generated: 4 quadrants (superior from 45° to 135°, inferior from 225° to 315°, nasal from 315° to 45°, and temporal from 135° to 225°), 12 clock-hours and average thickness (clock-hour 1 from 75° to 45°, clock-hour 2 from 45° to 15°, clock-hour 3 from 15° to 345°, clock-hour 4 from 345° to 315°, clock-hour 5 from 315° to 285°, clock-hour 6 from 285° to 255°, clock-hour 7 from 255° to 225°, clock-hour 8 from 225° to 195°, clock-hour 9 from 195° to 165°, clock-hour 10 from 165° to 135°, clock-hour 11 from 135° to 105°, and clock-hour 12 from 105° to 75°) and average thickness. All RNFLT hour measurements were aligned according to the orientation of the right eye. Hence, clock-hour 3 of the circumpapillary scan represented the nasal side of the optic disc for both eyes. We excluded all poor-quality scans analyzed at printouts with incorrect identification of the vitreoretinal surface. Only well-centered scans, with signal strength greater than 6, were included in our analysis. All images were acquired with undilated pupils by a single, well-trained ophthalmologist (Fernanda Cremasco), who was masked to the patient's diagnosis.

**Figure 1:**
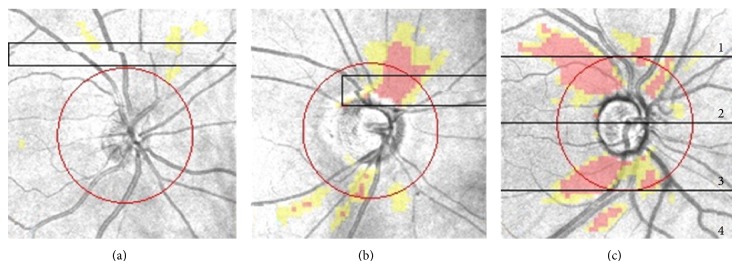
(a) Complete misalignment with vessel displacement of the complete scan line. Note that the misalignment occurs in the nasal direction. (b) Partial misalignment with vessel displacement of the scan. In this case, the misalignment occurs in the temporal direction. (c) Sectors used to classify the site of MAs.

MAs were defined as improper alignment of the vertical vessels in the PDF printout file. To better detect the presence of MAs, the SD-OCT scan (en face image) was enlarged to fit the screen (100% zoom). MAs were classified by two examiners (Kleyton A. Barella and Fernanda Cremasco) as partial (PMA), when they affected only part of the scanning line, and complete (CMA), when the entire line was affected. We also classified the site of the MA in 4 sectors, as illustrated in Figure 1(c).

We compared 12 clock-hour, global, and quadrant RNFLT between scans with MAs (CMAs + PMAs) and scans without MAs. We then investigated eyes with MAs situated in the superior area (sectors 1 and 2) and compared their RNFLT measurements with the scans without MAs. The same analysis was undertaken for eyes with MAs situated in the inferior area (sectors 3 and 4) and scans of eyes with MAs within the measurement ring (sectors 2 and 3).

The direction of the MAs was classified in nasal, temporal, superior, and inferior according to the direction of the shift of the vertical vessels on the SD-OCT scan (en face image).

### 2.3. Statistical Analysis

Data were analyzed using SPSS v.15.0.1 (IBM Inc., Chicago, IL, USA).

A post hoc power analysis was performed to determine the power of the study to detect differences in RNFLT between aligned and misaligned tests, given our sample size and effect size. The sample size of 56 allowed us to detect a 2.6 *μ*m difference in RNFLT between the exams, with a standard deviation (SD) of 4 *μ*m, at a power of 97% for misalignment in any of the 4 sectors. A sample size of 29 with misalignments in sectors 1 and 2 allowed us to detect a 4.3 *μ*m difference (SD = 5.7 *μ*m) at a power of 0.79. A sample size of 25, with misalignments in sectors 3 and 4, gave us a power of 0.79 to detect an 8.7 *μ*m difference (SD = 10.8 *μ*m) and a sample size of 37 with misalignments in sectors 2 and 3 gave us a power of 0.97 to detect 1.5 *μ*m difference (SD = 2.3 *μ*m).

Normal distribution of data was evaluated by the Kolmogorov-Smirnov test (with 5% significance level). RNFLT parameters and signal strength were analyzed using the paired Student's* t*-test. Wald-Wolfowitz runs test was applied to evaluate whether there was a tendency for misalignment in a particular direction. The* F*-test was employed to compare the percentage of change in RNFLT between normal and glaucomatous eyes. *P* values less than 0.05 were considered statistically significant.

## 3. Results

Fifty-six eyes of 56 patients were analyzed in this study, 27 of them were healthy eyes and 29 were glaucomatous. Of these, 40 eyes had a CMA and 16 eyes had a PMA.

The clinical characteristics of the study population are shown in Table 1. Overall, the mean age was 49.1 ± 13.5, 20 (36%) subjects were male, mean BCVA LogMAR equivalent was 0.04 ± 0.096, mean spherical equivalent was 0.25 ± 1.18 D, mean IOP was 14 ± 2.6 mmHg, and mean VF mean defect (MD) was −3.2 ± 5.4 dB.

Table 2 displays RNFLT measurements of SD-OCT exams with and without MAs (CMA or PMA) in any of the 4 sectors. There were no statistically significant differences in RNFLT measurements between aligned and misaligned exams.

Table 3 displays the mean SD-OCT parameters of exams with and without MAs (CMA or PMA) in the superior area (sectors 1 + 2). Mean SD-OCT measurements were not significantly different between aligned and misaligned exams, except for the 12 clock-hour, which showed lower mean RNFLT in misaligned exams compared to aligned exams (101.7 ± 39.7 *μ*m versus 111.4 ± 34.5 *μ*m, *P* = 0.043).

Table 4 displays mean SD-OCT parameters of exams with and without MAs (CMA or PMA) in the inferior area (sectors 3 + 4) and Table 5 displays the mean of SD-OCT parameters of exams with and without MAs (CMA or PMA) within the measurement ring (sectors 2 + 3). None of the SD-OCT parameters showed statistically significant differences between aligned and misaligned exams (Tables 4 and 5). In all previous comparisons, there were no statistically significant differences between signal strengths of eyes with and without MAs (*P* > 0.05).

A subgroup analysis was also performed in order to investigate whether exams with CMA would present more pronounced RNFLT measurements changes. Only exams with CMA (*n* = 40) were compared to aligned exams and the same results were observed: CMA exams in the superior area (sectors 1 + 2) showed lower mean RNFL thickness in the 12 clock-hour compared to aligned exams (*P* = 0.026).

It is important to consider that it is likely that eyes with thinner RNFLT may be more prone to the effect of MAs. When we applied the* F*-test, we found that the percentage change was similar in glaucomatous (*n* = 29) and normal eyes (*n* = 27) at the 12 clock-hour position (*P* = 0.094). This suggests that the effect of MAs is similar in normal and glaucomatous eyes.

Regarding the direction of the 56 misalignments, 1 MA was inferior, 5 MAs were superior, 14 MAs were temporal, and the majority of MAs (*n* = 36) were nasal (*P* = 0.020) (Figure 1(a)).

## 4. Discussion

Exams with MAs in the RNFLT deviation map have been excluded from OCTs studies, even without scientific evidence for this [12]. In this study we found that the presence of CMAs or PMAs on superior sectors (sectors 1 + 2) of the Cirrus SD-OCT scan (en face image) was associated with lower RNFLT in the 12 clock-hour (*P* = 0.043). This finding has clinical importance, since glaucomatous damage is frequently detected at the inferior and/or superior locations [12, 13]. Hence, the presence of misalignments on SD-OCT scans may affect the detection of RNFL damage in glaucoma patients. It is important to emphasize that the differences we observed can not be explained by differences in mean signal strength between eyes with and without MAs (Tables 2–5), which could potentially interfere with RNFLT measurements [14].

To our knowledge, this is the first study to assess the effects of single misalignments on RNFLT measurements, acquired on the same day from normal and glaucomatous patients. This is also the first study to analyze the direction of misalignments. Moreno-Montañés et al. [9] examined 26 patients with one aligned exam and one CMA exam obtained up to one month apart. They found an association between number of CMAs and increasing age. However, they found no statistical difference in global and quadrant RNFLT between aligned and misaligned exams. In fact, in accordance with their findings, when we compared RNFLT measurements between aligned and misaligned exams regardless of the location of the MA, we were not able to detect significant differences (Table 2). However, only when we analyzed the effects of superior MAs on superior RNFLT measurements differences were noticed. This confirms our hypothesis and is probably explained by the fact that the capture of the cube of 6 × 6 × 2 mm (200 lines) happens from the top to the bottom. Hence, it is possible that superior MAs do not affect RNFLT measurements obtained below that sector.

Vizzeri et al. [15] performed a similar study with the Stratus TD-OCT (Carl Zeiss Meditec Inc., Dublin, CA) and suggested that peripapillary scan circle misplacement secondary to motion artifacts produced significant changes in RNFL assessment: measurements for a given sector of the scan circle tended to increase for displacement toward the optic disc margin and to decrease when the displacement occurred in the opposite direction. Different from Stratus, the Cirrus OCT, used in our study, automatically places the measurement ring around the optic disc, reducing the errors of RNFL measurements caused by decentralization [16]. Recently, Taibbi et al. [17] evaluated the effect of scan circle displacements on RNFLT measurements generated from Cirrus^©^ SD-OCT scans with MAs passing through the optic disc. In this cross-sectional study, 70 scans from 18 healthy eyes and 100 scans from 26 glaucomatous eyes were divided into 85 pairs, each composed of a scan with one MA affecting the optic disc and a scan from the same eye without MA. They used an image registration technique to determine horizontal/vertical scan circle displacements. Interestingly, they found no difference in average and quadrant RNFLT between scans with and without motion artifacts. However, in healthy eyes, scans with motion artifacts had greater RNFLT in clock-hours 4 and 5 (*P* = 0.03), while in glaucomatous eyes scans with motion artifacts had greater RNFLT in clock-hours 4, 5, 10, and 11 (*P* < 0.05) and lower RNFLT in clock-hour 7 (*P* = 0.05), compared to scans without motion artifacts. This is not in accordance with our findings, since we found no statistical difference in scans with MAs passing through the measurement ring (Table 5). This difference may be due to the fact that Taibbi et al. [17] included patients with MAs passing through the optic disc, whereas we evaluated patients with MAs passing through the 3.46 mm diameter RNFL thickness measurement ring (sectors 2 + 3), an area greater than the optic disc (Figure 1(c)).

We also demonstrated that the direction of the misalignments occurs significantly more often to the nasal region (*P* = 0.020) (Figure 1(a)). Although eye movements may not be detectable during steady fixation, small movements, which are undetected visually but are commonly found in healthy persons [18], may explain MAs. One hypothesis is that the capitation lines, which are always located temporally to the fixation target (large green fixation asterisk), may cause these small eye movements in the nasal direction [19].

In 2006, Ishikawa et al. [20] proposed two solutions to improve image acquisition with OCTs: that the examination be carried out quickly and that an eye-motion tracking system be used to correct for eye movements. Image acquisition speed has improved with new SD-OCTs. Eye-tracking is used in some OCTs such as the Spectralis OCT (Heidelberg Engineering, Heidelberg, Germany) [21] and the new Cirrus HD-OCT 5000 (Carl Zeiss Meditec Inc., Dublin, CA), which features the FastTrac, that allows users to capture high-resolution B-scans in identical locations from visit to visit, and possibly eliminates motion artifacts during scan acquisition [16].

Our study has some limitations, such as being retrospective in nature, with a relatively small sample size (without statistical power to analyze healthy and glaucoma subgroups separately). Furthermore, we have not quantified the MAs, as performed by Taibbi et al. [17], which may interfere with RNFLT measurements. This may explain why MAs in the inferior sector or within the measurement ring were not found to cause significant changes in RNFLT measurements in the correspondent sectors.

## 5. Conclusions

Our study showed that CMAs or PMAs in the superior area (sectors 1 + 2) of the RNFL deviation thickness map of the SD-OCT lead to lower RNFLT measurements at the 12 clock-hour and that the direction of misalignment is usually to the nasal region. Further studies are needed using instruments equipped with eye-tracking to evaluate the occurrence of MAs during image acquisition.

## Figures and Tables

**Table 1:** Demographic characteristics of the eyes with complete or partial misalignments included in the study.

	Eyes (*n* = 56)
Age (years; mean ± SD)	49.1 (±13.5)
Gender (male [%] : female [%])	20 [36%]/36 [64%]
Race (Caucasian [%] : African-American [%])	48 [86%]/8 [14%]
Eye (right [%]; left [%])	49 [87%]/7 [13%]
Mean visual acuity (log MAR; mean ± SD)	0.04 (±0.096)
Health [%]; glaucoma [%]	27 [48%]/29 [52%]
Location of the MA (1/2/3/4)	14/16/22/4
Mean spherical equivalent (diopters; mean ± SD)	0.25 (±1.18)
Intraocular pressure (mmHg; mean ± SD)	14.0 (±2.6)
Medications (mean ± SD)	1.0 (±1.3)
MD (dB; mean ± SD)	−3.2 (±5.4)
PSD (dB; mean ± SD)	2.7 (±2.9)

SD = standard deviation; MA = misalignment; MD = mean deviation; dB = decibel; PSD = pattern standard deviation.

**Table 2:** Comparison of mean ± standard deviation (95% confidence interval) SD-OCT parameters between eyes with and without misalignments in any of the 4 sectors.

SD-OCT	AE (*n* = 56)	MAE (*n* = 56)	*P* value^†^
AT (*µ*m)	88.0 ± 14.3 (CI 59.9–116.0)	87.5 ± 15.3 (CI 57.5–117.5)	0.407
Quadrant, (*µ*m)			
Superior	109.1 ± 24.2 (CI 61.6–156.5)	106.9 ± 27.8 (CI 52.4–161.4)	0.197
Nasal	70.5 ± 10.3 (CI 50.3–90.6)	70.0 ± 12.6 (CI 43.3–94.7)	0.607
Inferior	109.1 ± 24.6 (CI 60.8–157.3)	109.6 ± 25.1 (CI 60.4–158.8)	0.712
Temporal	62.9 ± 10.9 (CI 41.5–84.2)	63.4 ± 12.1 (CI 39.7–87.1)	0.339
Clock-hour, (*µ*m)			
1	97.9 ± 26.0 (CI 46.9–148.8)	96.4 ± 30.8 (CI 36.0–156.8)	0.481
2	82.0 ± 14.5 (CI 53.5–110.4)	82.0 ± 17.4 (CI 47.9–116.1)	0.960
3	61.0 ± 10.8 (CI 39.8–82.1)	60.1 ± 13.5 (CI 33.6–86.6)	0.453
4	68.4 ± 12.6 (CI 43.7–93.0)	68.0 ± 13.6 (CI 41.3–94.7)	0.723
5	90.7 ± 22.9 (CI 45.8–135.5)	93.2 ± 27.2 (CI 39.9–146.5)	0.147
6	118.4 ± 31.8 (CI 56.0–180.7)	118.1 ± 32.3 (CI 54.8–181.4)	0.822
7	118.4 ± 31.9 (CI 55.8–180.9)	116.8 ± 29.8 (CI 58.4–175.1)	0.269
8	64.2 ± 14.1 (CI 36.5–91.8)	64.9 ± 17.3 (CI 31.0–98.8)	0.503
9	52.8 ± 14.0 (CI 25.3–80.2)	53.5 ± 14.2 (CI 25.7–81.3)	0.382
10	71.8 ± 14.0 (CI 44.3–99.2)	71.7 ± 13.6 (CI 45.0–98.4)	0.975
11	115.0 ± 29.5 (CI 57.1–172.8)	113.6 ± 28.8 (CI 57.2–170.0)	0.188
12	114.8 ± 34.7 (CI 46.7–182.8)	110.6 ± 39.1 (CI 34.0–187.2)	0.175
Signal strength	8.0 ± 0.7 (CI 6.6–9.3)	8.1 ± 0.7 (CI 6.7–9.5)	0.277

SD-OCT = spectral domain optical coherence tomography; AE = aligned exam; MAE = misaligned exam; AT = average thickness; CI = confidence interval. ^†^Student's *t*-test.

**Table 3:** Comparison of mean ± standard deviation (95% confidence interval) SD-OCT parameters between eyes with and without misalignments in the superior area (sectors 1 and 2).

SD-OCT	AE (*n* = 30)	MAE (*n* = 30)	*P* value^†^
AT (*µ*m)	85.2 ± 13.7 (CI 55.6–109.4)	84.2 ± 14.8 (CI 55.2–113.2)	0.351
Quadrant, (*µ*m)			
Superior	106.3 ± 24.8 (CI 57.7–154.9)	101.7 ± 28.9 (CI 45.1–158.3)	0.084
Nasal	69.3 ± 7.8 (CI 54.0–84.6)	68.0 ± 11.7 (CI 45.1–90.9)	0.444
Inferior	104.1 ± 22.2 (CI 60.6–147.6)	105.6 ± 22.5 (CI 61.5–149.7)	0.412
Temporal	61.0 ± 11.0 (CI 39.4–82.6)	61.7 ± 11.1 (CI 39.9–83.5)	0.145
Clock-hour, (*µ*m)			
1	94.3 ± 26.5 (CI 42.4–146.2)	89.7 ± 29.6 (CI 37.6–141.8)	0.155
2	80.2 ± 11.0 (CI 58.5–101.7)	79.7 ± 16.1 (CI 48.1–111.3)	0.814
3	59.5 ± 9.1 (CI 41.7–77.3)	58.2 ± 14.7 (CI 29.4–87.0)	0.531
4	67.8 ± 11.8 (CI 44.7–90.9)	66.4 ± 11.3 (CI 44.3–88.5)	0.385
5	85.8 ± 20.8 (CI 45.0–126.6)	87.0 ± 20.4 (CI 47.0–127.0)	0.510
6	111.8 ± 30.1 (CI 52.8–170.8)	114.4 ± 30.0 (CI 55.6–173.2)	0.327
7	114.9 ± 32.4 (CI 51.5–178.5)	115.4 ± 30.9 (CI 54.8–176.0)	0.753
8	62.5 ± 13.1 (CI 36.8–8.2)	62.7 ± 13.2 (CI 36.8–88.6)	0.876
9	50.2 ± 8.3 (CI 34.9–66.5)	51.6 ± 9.7 (CI 32.6–70.6)	0.050
10	70.4 ± 16.6 (CI 37.9–102.9)	70.9 ± 16.3 (CI 39.0–102.8)	0.548
11	113.4 ± 30.1 (CI 54.4–172.4)	113.4 ± 30.2 (CI 4.2–172.6)	0.979
12	111.4 ± 34.5 (CI 43.8–179.0)	101.7 ± 39.7 (CI 23.9–179.5)	**0.043^+^**
Signal strength	8.1 ± 0.7 (CI 6.7–9.5)	8.2 ± 0.6 (CI 7.0–9.4)	0.255

SD-OCT = spectral domain optical coherence tomography; AE = aligned exam; MAE = misaligned exam; AT = average thickness; CI = confidence interval. ^+^ *P* value <0.05. ^†^Student's *t*-test.

**Table 4:** Comparison of mean ± standard deviation (95% confidence interval) SD-OCT parameters between eyes with and without misalignments in the inferior area (sectors 3 and 4).

SD-OCT	AE (*n* = 26)	MAE (*n* = 26)	*P* value^†^
AT (*µ*m)	91.1 ± 14.6 (CI 62.5–119.7)	91.2 ± 15.3 (CI 61.2–121.1)	0.859
Quadrant, (*µ*m)			
Superior	112.5 ± 23.6 (CI 66.2–158.8)	112.9 ± 25.8 (CI 62.3–163.4)	0.830
Nasal	72.0 ± 12.7 (CI 47.1–96.9)	72.3 ± 13.5 (CI 45.8–98.7)	0.746
Inferior	114.8 ± 26.5 (CI 64.8–164.8)	114.3 ± 27.5 (CI 60.4–168.2)	0.838
Temporal	65.1 ± 10.7 (CI 44.1–86.1)	65.3 ± 13.1 (CI 39.6–90.9)	0.867
Clock-hour, (*µ*m)			
1	102.0 ± 25.3 (CI 52.4–151.6)	104.1 ± 30.9 (CI 43.5–164.6)	0.455
2	84.0 ± 17.6 (CI 49.5–118.5)	84.7 ± 18.8 (CI 47.9–121.5)	0.689
3	62.6 ± 12.5 (CI 38.1–87.1)	62.2 ± 11.8 (CI 39.1–85.3)	0.682
4	69.1 ± 13.7 (CI 42.2–96.0)	69.8 ± 15.8 (CI 38.8–100.7)	0.616
5	96.3 ± 24.3 (CI 48.7–143.9)	100.5 ± 32.2 (CI 37.2–163.8)	0.201
6	126.1 ± 32.5 (CI 62.4–189.8)	124.1 ± 34.7 (CI 56.1–192.1)	0.529
7	122.3 ± 31.4 (CI 60.8–183.8)	118.3 ± 28.9 (CI 61.7–174.9)	0.111
8	66.1 ± 15.3 (CI 36.1–96.1)	67.5 ± 21.1 (CI 26.1–108.8)	0.492
9	55.9 ± 18.2 (CI 20.2–91.6)	55.6 ± 18.0 (CI 20.3–90.8)	0.819
10	73.3 ± 10.3 (CI 53.1–93.5)	72.7 ± 9.8 (CI 53.5–91.9)	0.450
11	116.8 ± 29.2 (CI 59.6–174.0)	113.8 ± 27.7 (CI 59.5–168.0)	0.100
12	118.6 ± 35.2 (CI 49.6–187.6)	120.9 ± 36.6 (CI 49.2–192.6)	0.514
Signal strength	8.0 ± 0.7 (CI 6.6–9.4)	8.1 ± 0.8 (CI 6.5–9.6)	0.646

SD-OCT = spectral domain optical coherence tomography; AE = aligned exam; MAE = misaligned exam; AT = average thickness; CI = confidence interval. ^†^Student's *t*-test.

**Table 5:** Comparison of mean ± standard deviation (95% confidence interval) SD-OCT parameters between eyes with and without misalignments within the measurement ring (sectors 2 and 3).

SD-OCT	AE (*n* = 38)	MAE (*n* = 38)	*P* value^†^
AT (*µ*m)	88.5 ± 14.5 (CI 60.1–116.9)	88.6 ± 15.1 (CI 59.0–118.2)	0.890
Quadrant, (*µ*m)			
Superior	108.3 ± 21.0 (CI 67.1–149.5)	107.8 ± 23.9 (CI 61.0–154.6)	0.735
Nasal	71.7 ± 11.0 (CI 50.1–93.3)	72.4 ± 11.4 (CI 50.1–94.7)	0.383
Inferior	110.4 ± 26.2 (CI 58.5–162.3)	110.8 ± 26.6 (CI 58.7–162.9)	0.831
Temporal	63.3 ± 12.2 (CI 39.4–87.2)	63.5 ± 13.6 (CI 38.6–92.0)	0.844
Clock-hour, (*µ*m)			
1	96.1 ± 23.3 (CI 50.4–141.8)	97.2 ± 27.8 (CI 42.7–151.7)	0.601
2	83.8 ± 16.5 (CI 51.0–115.6)	84.6 ± 17.2 (CI 50.9–118.3)	0.583
3	62.7 ± 11.2 (CI 40.7–84.7)	62.9 ± 11.1 (CI 41.1–84.7)	0.821
4	68.5 ± 11.9 (CI 45.2–91.8)	69.7 ± 13.7 (CI 42.8–96.6)	0.301
5	92.3 ± 23.1 (CI 47.0–137.6)	95.8 ± 29.3 (CI 38.4–153.2)	0.122
6	122.2 ± 34.3 (CI 55.0–189.4)	121.7 ± 35.4 (CI 52.3–191.1)	0.813
7	116.9 ± 32.3 (CI 53.6–180.2)	114.8 ± 29.2 (CI 57.6–172.0)	0.262
8	64.1 ± 14.9 (CI 34.9–93.3)	64.6 ± 19.2 (CI 27.0–102.2)	0.716
9	53.3 ± 16.1 (CI 21.7–84.9)	53.5 ± 16.3 (CI 21.6–85.4)	0.868
10	72.6 ± 14.8 (CI 43.6–101.6)	72.2 ± 14.3 (CI 44.2–100.2)	0.656
11	116.5 ± 29.0 (CI 59.7–173.3)	114.7 ± 28.9 (CI 58.1–171.3)	0.185
12	112.3 ± 27.1 (CI 59.2–165.4)	111.3 ± 32.3 (CI 48.0–174.6)	0.711
Signal strength	8.0 ± 0.7 (CI 6.6–9.4)	8.1 ± 0.8 (CI 6.5–9.7)	0.378

SD-OCT = spectral domain optical coherence tomography; AE = aligned exam; MAE = misaligned exam; AT = average thickness; CI = confidence interval. ^†^Student's *t*-test.
